# Study on Rheological Properties of Graphene Oxide/Rubber Crowd Composite-Modified Asphalt

**DOI:** 10.3390/ma15186185

**Published:** 2022-09-06

**Authors:** Zhenxia Li, Tengteng Guo, Yuanzhao Chen, Luochao Dong, Qi Chen, Menghui Hao, Xu Zhao, Jinyuan Liu

**Affiliations:** 1 School of Civil Engineering and Communication, North China University of Water Resources and Electric Power, Zhengzhou 450045, China; 2 Henan Province Engineering Technology Research Center of Environment Friendly and High-Performance Pavement Materials, Zhengzhou 450045, China; 3 Zhengzhou City Key Laboratory of Environmentally Friendly High Performance Road and Bridge Materials, Zhengzhou 450045, China; 4 Henan Communications Planning & Design Institute Co., Ltd., Zhengzhou 450046, China

**Keywords:** pavement material, graphene oxide, rubber powder, rheological properties at high and low temperatures, micro-analysis

## Abstract

In order to improve the durability of asphalt pavement and prolong the service life of heavy traffic asphalt pavement, graphene oxide (GO) and rubber powder (RP) were used as composite modifiers to modify matrix asphalt. The high-temperature rheological properties of composite-modified asphalt at different temperatures and frequencies were analyzed. The influence of different stress levels on the viscoelastic properties of composite-modified asphalt was evaluated. The low-temperature rheological properties of composite-modified asphalt were studied. The difference between RP-modified asphalt and GO/RP composite-modified asphalt was analyzed, and the mechanism of GO and RP on asphalt modification was explored. The results show that the composite-modified asphalt has good rheological properties at low temperature, relatively smooth surface and stable network structure, which improves the segregation problem of RP in matrix asphalt. At present, GO and RP are rarely used as composite modifiers to modify matrix asphalt at home and abroad, which is an innovation in material selection.

## 1. Introduction

With the rapid development of road engineering technology in China, the total mileage of highway traffic has also been increasing. By the end of 2021, the total mileage of highway in China exceeded 5.2 million kilometers [1]. The traditional asphalt pavement is prone to road diseases, such as deformation of the road rut, caused by the poor stability performance of the mixture at high temperature, and the lack of low-temperature performance will lead to cracks in the road surface, accompanied by many problems such as pits and loose material, resulting in a low service life and high maintenance cost of the road surface. This is also one of the main difficulties faced by the development of our country′s transportation field in the new era. Therefore, improving the safety and durability of asphalt pavement has become a new research direction.

The modification of base asphalt with rubber powder (RP) can not only improve the rutting resistance of asphalt, but also significantly enhance the low-temperature characteristics of asphalt [2,3]. RP-modified asphalt has better high-temperature rheological properties, and with the increase in rubber powder content, its high-temperature rheological properties also tend to improve [4]. Rheological theory is an effective method to study the compatibility of rubber powder with base asphalt [5]. Warming agent can enhance the high-temperature performance of asphalt. The G* (complex modulus) of asphalt added with warming agent shows an increasing trend, the phase angle δ shows a decreasing trend, and the improved rutting factor G*/(sinδ) increases. The high-temperature deformation resistance of surfactant (SDYK) type warm stirring powder (WCR)-modified asphalt increases first and then decreases, and the high-temperature deformation resistance of viscosity reducer (EM) WCR-modified asphalt gradually decreases [6]. Rubber powder can significantly improve the high-temperature performance of the base asphalt. When using lower rubber fineness or lower grade pure asphalt to produce rubber-modified asphalt, the cumulative strain is significantly reduced; Using Jnr and R index can better characterize the anti-rutting performance of different rubber powder modified asphalt. [7].

GO can significantly improve the deformation resistance of matrix asphalt at high temperature [8,9,10,11]. Since GO is a layered structure and has its own superior strength and stiffness, the rheological properties of GO to asphalt under the action of temperature and load has an improvement effect [12]. The addition of GO can change the ratio between the original components of asphalt, increase the ratio of asphaltenes and colloids, and improve its colloidal stability [13]. GO is stably dispersed in asphalt, and by forming intercalation junctions, it can improve the high-temperature properties and heat aging resistance of asphalt but cannot improve the ability of asphalt to resist cracking [13]. GO has a significant contribution to the ability to resist rutting deformation, and GO can be perfectly distributed in the matrix asphalt, and the compatibility with asphalt is relatively good [14]. Graphene oxide was mixed with bromobutane to synthesize butylated graphene oxide composite material, and then compounded with styrene-butadiene-styrene (SBS) to prepare C_4_H_9_-GO/SBS-modified asphalt, which was mixed with 5% SBS-modified asphalt. C_4_H_9_-GO/SBS-1.0-modified asphalt showed the best performance, the softening point increased by 11.4%, the ductility increased by 19.3%, and the permeability decreased by 10.2%; at 1.0% C_4_H_9_-GO content, C_4_H_9_-GO/SBS composite modified bitumen exhibited the best high-temperature performance and lower stress sensitivity [15]. GO can improve the UV aging resistance of 90A and SBS maleic anhydride (MA) [16]. GO can improve the high-temperature properties of asphalt, but the changes in low-temperature properties are not significant; GO can significantly improve the rutting factor, failure temperature, viscoelastic properties, and high-temperature plastic deformation resistance of asphalt [17]. The addition, GO can significantly improve the anti-aging ability of SBS-modified asphalt. For GO composite SBS-modified asphalt, GO improves the high-temperature properties of modified asphalt, but weakens the low-temperature properties. After thermal oxygen aging and UV aging, GO composite SBS-modified asphalt G* increases at high temperature, δ and strain recovery rate R values decrease, creep stiffness increases at low temperature, and stress relaxation ability decreases [18]. When the GO content is 0.6%, the ductility and softening point of SBS-modified asphalt are increased by 26% and 8.3%, respectively, and GO composite SBS-modified asphalt has better high- and low-temperature performance [19].

To sum up, GO can be stably dispersed in asphalt, and the modification of asphalt is to improve the anti-ultraviolet aging performance of asphalt. The stress-sensitive properties are reduced, but the low-temperature effect on the base asphalt is not improved significantly. Base asphalt with RP has lower penetration, higher softening point and viscosity, thus enhancing high-temperature performance. The addition of RP can also reduce the sensitivity of the base asphalt to temperature changes, improve its flexibility at low temperature, and enhance its ability to resist cracking at low temperatures. However, RP particles have the problem that they are not very compatible with the matrix asphalt, and the RP particles have a segregation phenomenon in the matrix asphalt, resulting in poor storage stability. Therefore, in this paper, GO and RP are used as composite modifiers to modify matrix asphalt, in order to compensate each other for their shortcomings as a single admixture, and to comprehensively evaluate the rheological properties and viscoelastic properties of composite-modified asphalt at high and low temperatures. In the current research results, GO and RP are rarely used as composite modifiers to modify the matrix asphalt at home and abroad, and there are few related literature reports, which has great advantages for the road performance of GO/RP composite modified asphalt.

## 2. Materials

### 2.1. Raw Materials Preparation

#### Asphalt

The asphalt selected in this paper is A-grade 70# asphalt provided by Zhengzhou Zhengfa Municipal Construction Co., Ltd. (Zhengzhou, China), and its basic properties are measured according to the relevant test procedures [20]. The test results are shown in Table 1.

### 2.2. Composite Preparation

#### 2.2.1. Rubber Powder

The test uses rubber powder (RP) with a fineness of 40 mesh, a density of 1.12 g/cm^3^, and a moisture content of 0.55%.

#### 2.2.2. Graphene Oxide

Graphene oxide (GO) is an oxide of graphene. The introduction of oxygen-containing groups makes it more active and easy to functionalize, so that graphene oxide has chemical stability, which provides a surface modification active position and large specific surface area for the synthesis of composite materials. In this experimental study, GR-005-4 high-quality industrial grade graphene oxide (GO) produced by Suzhou Carbonfeng Graphene Technology (Suzhou, China) was used. The basic performance indicators are shown in Table 2.

## 3. Experimental

### 3.1. Preparation of Graphene Microsheet/Rubber Powder Composite-Modified Asphalt

The temperature of the electric heating blast drying oven was adjusted to 180 °C, then 300 g of base asphalt was placed into it to dehydrate it for later use, and at the same time the rubber powder modifier was inserted into the electric heating blast drying oven with a temperature of 100 °C for 60 min to dry it. Afterwards, 0.06% GO and 16% rubber powder were weighed for use in subsequent experiments. The temperature of base asphalt was raised to 175 °C. GO, which had been weighed, was first added, and rubber powder was added in batches after high-speed shearing for 10 min. Manual stirring was used to speed up the dispersion, and the shear rate was maintained at 5000 rpm and at 180~190 °C for 50 min. The prepared modified asphalt was placed in an electric heating blast drying oven at 180 °C for 30 min to swell and develop, and the air mixed in the asphalt was removed by stirring with a glass rod every 10 min, and the preparation of GO/RP composite-modified asphalt was completed.

### 3.2. Dynamic Shear High-Temperature Rheological Test

In order to more systematically analyze the viscoelastic properties of GO/RP composite-modified asphalt at high temperature, according to the Strategic Highway Research Program (SHRP) plan of the United States, the rheological properties of composite-modified asphalt were studied by dynamic shear rheometer (DSR) (Zhengzhou, China). The rheological properties were automatically tested, and output data were acquired by computer according to the specification, and the original data were analyzed by different means to achieve the conclusion. The yield stress and plastic viscosity involved in the test process were automatically completed by the computer. The complex modulus G*, phase angle δ and rutting factor G*/sinδ of asphalt samples were analyzed by different loading frequencies. In this paper, DHP-1 type dynamic shear rheometer is used to study the high-temperature rheological properties of asphalt. In order to increase the stability of the test results, the test temperature range in this section was selected between 46 °C and 88 °C. The parallel plate fixture specification and plate spacing were unified with a diameter of 25 mm and a thickness of 1 ± 0.05 mm. When the strain control method was adopted, the appropriate strain value of the composite-modified asphalt was selected to be 1% for the test. The dynamic shear rheometer can automatically control the strain without the operator’s adjustment. When the temperature reaches equilibrium, the equipment automatically tested at a frequency of 10 rad/s and a selected stress (or strain) target value. The angular frequency of the temperature scan was 10 rad/s, and the frequency range of the frequency scan was 0.1–100 rad/s. When performing temperature scanning on base asphalt, RP-modified asphalt and GO/RP-modified asphalt, the complex shear modulus of base asphalt, RP-modified asphalt and GO/RP-modified asphalt were analyzed by using the temperature scanning form in the DSR test. The development trend of G* and phase angle δ at different temperatures, the rutting factor–temperature curve equation was fitted according to the rutting factor test results, and the slope |A| value was used to study the temperature sensitivity of asphalt [21]. In frequency scanning, the time–temperature equivalence principle was applied to the test results, and the viscoelastic principal curve was drawn by fitting and translating the complex modulus–angular frequency curve under five temperature environments, and the variation rules of three kinds of asphalt G* were compared and evaluated.

### 3.3. Multiple Stress Creep and Recovery Test

Modified asphalt is a typical viscoelastic material with creep and deformation recovery characteristics. The multiple stress creep and recovery test (MSCR) test can reflect the cumulative deformation of asphalt under repeated unidirectional loading and unloading. Viscoelastic response and rut resistance of asphalt can be reflected by measuring the unrecoverable creep compliance (Jnr) and strain recovery rate (R) of asphalt. The MSCR test is a process of repeated loading and unloading. The test contains 20 creep cycles, and each creep cycle lasts for 10 s, a total of 200 s. At the beginning of each cycle, the sample was loaded with a specified shear stress for 1 s, and then unloaded for 9 s. In this study, two stress levels of 0.1 kPa and 3.2 kPa were selected in the MSCR test, which was mainly used to simulate the two load conditions of light and heavy traffic. It was also determined according to the modified asphalt multi-stress creep recovery (MSCR) test procedure. The creep recovery ability of asphalt under different loads was evaluated, and then the cycle process of repeated rolling to recovery and rolling of asphalt pavement was simulated in practical application.

During each cycle, bitumen samples underwent two stages of creep and recovery. During the loading process, the strain started to rise from the initial strain γ_0_ to the peak value γ_p_, and during the unloading process, the strain began to decrease gradually. The residual strain value after unloading for 9 s is γ_nr_, and the reduced strain value during unloading is γ_r_. The strain recovery rate R is expressed as the ratio of the recoverable strain to the strain peak value, which represents the elastic recovery ability of the asphalt after deformation. J_nr_ is the ratio of the unrecoverable strain amount to the test loading stress, which reflects the strength of the anti-deformation performance of the asphalt.

When the load is 0.1 kPa, the γ_r_(0.1, N) under a single loading cycle is as Formula (1):

(1) $$\gamma_{r}\left(0.1,N\right)=\frac{\gamma_{r}}{\gamma_{p}}\times 100\;$$

J_nr_ (0.1, N) under a single loading cycle is as Formula (2):

(2) $$J_{nr}\left(0.1,\;N\right)=\frac{\gamma_{nr}-\gamma_{0}}{0.1}$$

In the formula: N—the number of loading cycles, ranging from 1 to 10; 0.1—the shear stress, in kPa.

Similarly, the values of γ_r_ (3.2, N) and J_nr_ (3.2, N) at a shear stress of 3.2 kPa under a single loading cycle can be obtained, and the final irreversible creep compliance (J_nr_) and strain recovery rate (R) are obtained by calculating 10 loading-unloading cycles. In this section, the MSCR sample size is 25 mm in diameter ×1 mm in thickness, and the test temperature is 64 °C.

### 3.4. Low-Temperature Bending Rheological Test

The low-temperature cracking resistance of asphalt pavement is mainly determined by the low-temperature performance of asphalt binder. At present, it is considered that the low-temperature bending rheological test (BBR) proposed by American SHRP is the most accurate [22]. In this paper, the Advanced Thermal Solutions (ATS) low-temperature bending rheometer produced in the United States is used for experimental research. Trabecular bending specimens of matrix asphalt, RP-modified asphalt and GO/RP-modified asphalt were prepared. The size of the specimens was 127 mm × 12.7 mm × 6.35 mm. The specimens were continuously loaded under certain stress at low temperatures of −12 °C, 1–8 °C and −24 °C, respectively, for 240 s. During the test, the computer automatically collected the creep stiffness S value and creep rate M value of the trabecular specimen at 15 s, 30 s, 60 s, 120 s and 240 s through the sensor. According to SHRP requirements, S ≤ 300 MPa and M ≥ 0.3 during 60 s; the stress relaxation time after deformation was further analyzed by Burgers model, and the creep properties at low temperature were evaluated comprehensively.

### 3.5. Test of SEM

In this experiment, a Gemini Sigma 300 scanning electron microscope (SEM) (Zhengzhou, China) was used to scan the modified asphalt specimen. First, the sample was fixed on the sample plate with conductive adhesive, and a layer of conductive heavy metal particles was plated on the surface of the sample under vacuum conditions. The dimensions of the test specimens are all within the size range of 10 mm × 10 mm × 5 mm. The SEM morphology analysis of matrix asphalt, rubber powder-modified asphalt and GO/rubber powder composite-modified asphalt was carried out. The magnification of matrix asphalt was 100 times, and the magnification of rubber powder-modified asphalt and GO/rubber powder composite-modified asphalt were 500 times.

### 3.6. Test of Fourier Infrared Spectrum

Frontier Fourier infrared spectrometer was used for infrared spectrum test scanning analysis. The wave number ranges from 4000–400 cm^–1^, the resolution is 4 cm^–1^, and the scanning times are 32. The matrix asphalt, rubber powder-modified asphalt and graphene oxide/rubber powder composite-modified asphalt were tested by infrared spectroscopy. The samples were tested by Attenuated Total Refraction (ATR) method without special sample preparation. During analysis, the location, number and intensity of absorption peaks can be determined. Thus, the molecular structure and group types of substances can be identified [23]

## 4. Test Results and Analysis

### 4.1. Dynamic Shear Rheological Test Analysis

#### 4.1.1. Temperature Sweep

**Figure 1:**
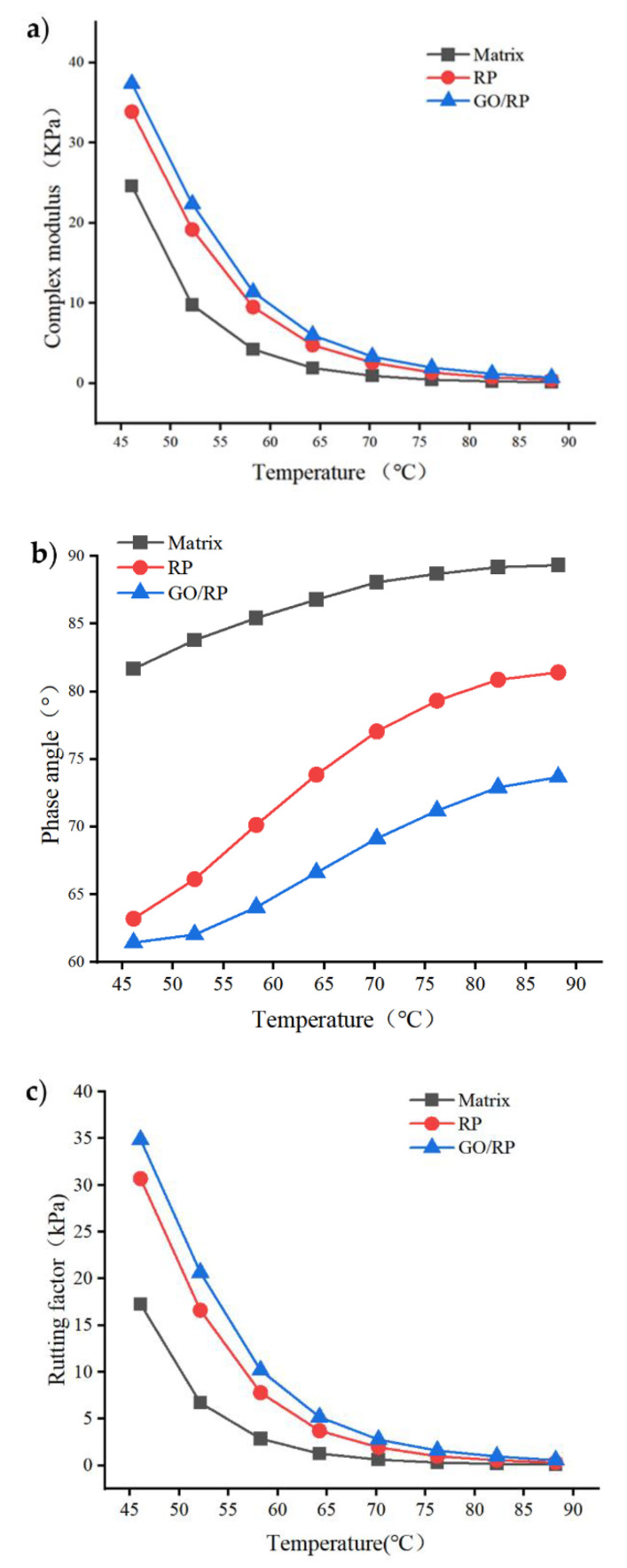
Temperature scanning test results: (**a**) complex modulus test results; (**b**) phase angle test results; (**c**) rutting factor temperature curves of three kinds of asphalt.

It can be seen from Figure 1a that the complex modulus G* of the three kinds of asphalt shows a decreasing trend with the increase in temperature. Among them, the complex modulus G* changes most obviously in the range of 45–55 °C. At the same temperature, the G* quantity relationship of three kinds of asphalt is GO/RP composite-modified asphalt > RP-modified asphalt > matrix asphalt. It shows that the addition of nano GO improves the physical compatibility between RP and matrix asphalt. GO and RP form a stable structure, so that RP is uniformly dispersed in matrix asphalt. Therefore, the high-temperature performance of GO/RP composite-modified asphalt is better than RP-modified asphalt and base asphalt.

The phase angle δ is used to characterize the proportion of the non-recoverable part of the material. For the material with great elasticity, its δ is close to 0°, on the contrary, the δ of the viscous material is almost 90 °C. It can be seen from Figure 1b that when the temperature is between 46 °C and 88 °C, the phase angles of the three pitches increase gradually with the increase in temperature. At the same temperature, the magnitude relationship of δ is matrix asphalt > RP-modified asphalt > GO/RP composite-modified asphalt, indicating that GO/RP composite-modified asphalt has better elastic properties and resistance to permanent deformation at high temperature.

It can be seen from Figure 1c that the change trend of rutting factor–temperature curve of three original samples of asphalt is basically the same as that of complex modulus –temperature. The larger the G*/sinδ value of asphalt is, the stronger the high-temperature plastic deformation resistance of asphalt is. Both G* and G*/sinδ indicate the shear deformation resistance of asphalt. With the increase in temperature, the three kinds of asphalt will eventually lose elasticity, so the values tend to be basically the same, but at low temperature, GO has improved the shear deformation resistance of RP-modified asphalt. At the same temperature, the G*/sinδ value of the GO/RP composite-modified asphalt is the highest, and the G*/sinδ value of the base asphalt is the lowest. The improvement of the rutting factor can also indicate that the superior strength of nano-GO itself and RP form a unified system, which improves the overall strength of the asphalt. The high mechanical strength of GO and the better elastic properties of RP can effectively prevent the deformation of asphalt when added as a modifier to asphalt. This is of great significance to the anti-fatigue failure ability of asphalt in high-temperature environments. 

In order to display the temperature sensitivity of the three asphalts more intuitively, the logarithm of the above rutting factor data is now used for linear fitting, and the fitting expression is as shown in Equation (3).

(3) $$1g\left(\frac{G^{*}}{sin\delta }\right)=A\cdot T+B$$

The fitting results are summarized as shown in Table 3.

**Figure 2:**
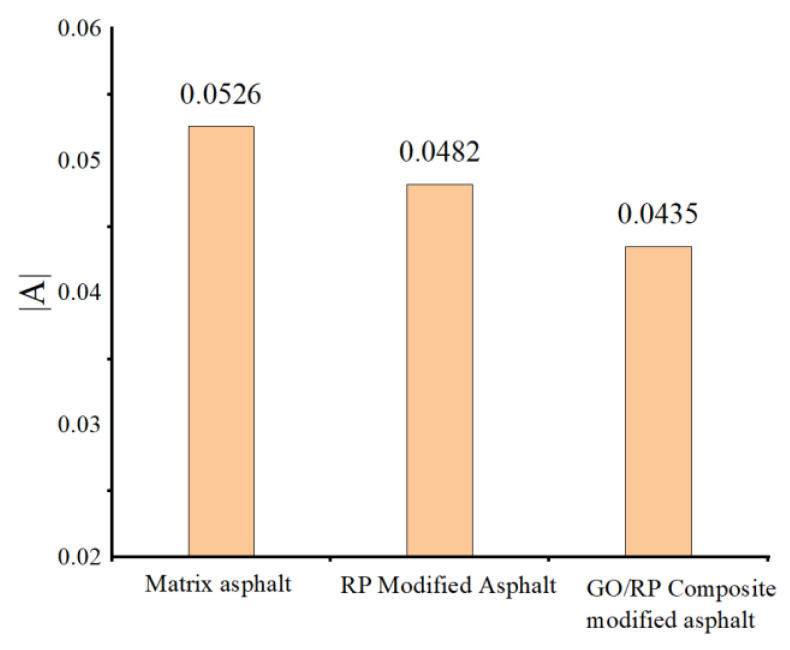
Three original asphalt |A| values.

The rheological properties are automatically tested and output data by computer according to the specification, and the original data are analyzed by different means to reach the conclusion. The yield stress and plastic viscosity involved in the test process are automatically completed by computer. It can be seen from Table 3 that the constants A < 0, B > 0, and the coefficient A can be regarded as the slope of the linear equation after fitting. The larger the value of |A|, the more easily the asphalt is affected by temperature changes, that is, the poor temperature stability. The fitted slope |A| is shown in Figure 2.

In order to more intuitively express the superiority of GO after adding, the fitting observation is carried out. From Figure 2, it can be seen that the maximum slope |A| value in the rutting factor fitting function is matrix asphalt, and the minimum is GO/RP composite-modified asphalt. The fitting results also confirm that compared with GO/RP composite, matrix asphalt is more prone to high-temperature deformation than the former. At this time, the high temperature is about 90 °C, and the opening conditions of 90 °C will not be encountered in practical engineering applications. When the temperature is low, the shear deformation resistance of GO to RP-modified asphalt is improved, indicating that the matrix asphalt has the highest sensitivity to temperature in practical engineering applications. High-temperature deformation is more likely to occur. The sensitivity of GO/RP composite-modified asphalt to temperature is lower than that of RP-modified asphalt, which reflects that GO/RP composite-modified asphalt has better temperature stability, indicating that the addition of GO can better improve the high-temperature performance of RP-modified asphalt.

#### 4.1.2. Frequency Sweep

1. Frequency sweep test

**Figure 3:**
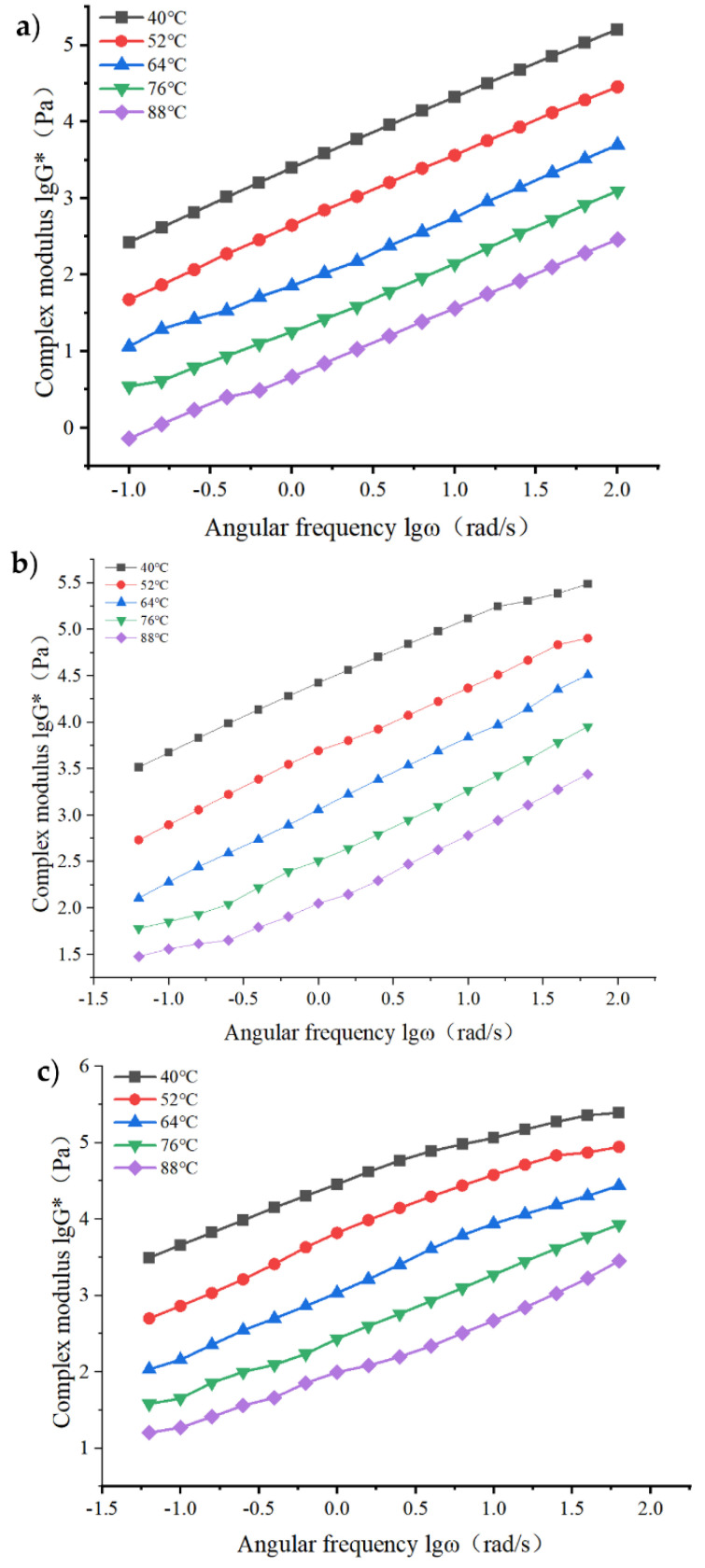
Frequency scanning test results: (**a**) variation curve of complex modulus of matrix asphalt; (**b**) variation curve of complex modulus of matrix asphalt; (**c**) variation curve of complex modulus of GO and rubber powder composite-modified asphalt.

For asphalt materials, the dynamic response characteristics of asphalt materials under different shear frequencies can be analyzed more comprehensively through frequency sweep tests. In this section, the selected test temperature is between 40 °C and 88 °C with 12 °C as the temperature interval, and DSR tests are used to study the G* of three asphalts with different frequencies (0.1–100 rad/s) in five temperature environments. After the complex modulus and angular frequency data are processed accordingly, the regular diagram of the variation of lgG* with lgω is drawn. According to the difference of the complex modulus of different asphalts at a certain temperature, the rheological properties before and after modification are analyzed. When strain control mode is adopted, the appropriate strain value of composite-modified asphalt is 1%. The dynamic shear rheometer can automatically control the strain without operator adjustment, and has been checked to be in the linear viscoelastic range. The test results are shown in Figure 3.

It can be seen from Figure 3 that under the same temperature conditions, the G* of the three asphalts all show a trend of increasing with the increase of ω. When the temperature is between 40 °C and 88 °C the rate of change of the complex modulus is basically the same, and the G* values of the three asphalts all decrease with the increase in temperature, indicating that the temperature has a great influence on the asphalt. The increase in temperature leads to the slow softening of asphalt, and the elastic components in asphalt gradually decrease, which makes the physical properties of asphalt change from viscoelasticity to ideal viscous fluid, and its G* becomes smaller. The test results show that the rise of pavement temperature weakens the ability of asphalt to resist high-temperature deformation, leading to the gradual increase in road rut.

2. Principal curve analysis of frequency sweep results

In this section, the displacement factor of the fitting curve of three kinds of original asphalt at the selected temperature is calculated by fitting the double logarithm of the complex modulus–angular frequency. Finally, the viscoelastic main curve is constructed by using the time–temperature equivalence principle to increase the analysis range [24,25,26].

3. Determination of displacement factor

Firstly, the frequency scanning results of matrix asphalt, RP-modified asphalt and GO/RP composite-modified asphalt were fitted by double logarithmic curve equation. The results are shown in Table 4.

In this excerpt, G* = 1 kPa, that is, lgG* = three times into the fitting equation in Table 4, the corresponding lgω value is calculated, the temperature of 40 °C referenced as the bench-mark, and the angular frequency displacement factor discovered under other temperatures, the results are shown in Table 5.

4. Asphalt main curve analysis

**Figure 4:**
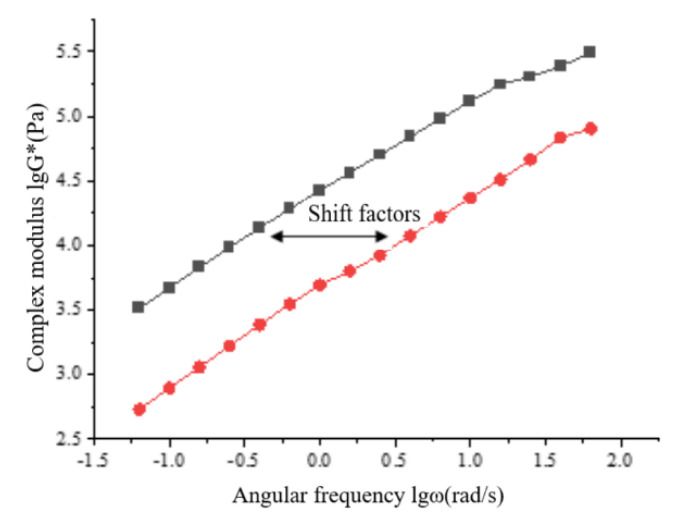
Schematic diagram of displacement factor (Black line: the lgG*-lgώ curve of the frequency sweep test of the asphalt sample at 40 °C, which is defined as the reference temperature line. Red line: lgG*-lgώ curve of asphalt sample at other temperatures except the reference temperature line).

The isothermal aging conversion method is to first select a certain temperature as the reference temperature, and move the curves at other temperatures horizontally in the horizontal direction to the distance of a displacement factor to the temperature in the horizontal direction, so as to achieve the purpose of basically overlapping the two curves. Finally, it will be superimposed into a lgG * -lgω curve in a wider temperature region, that is, the viscoelastic master curve. The time–temperature equivalence principle can be used to evaluate the influence of temperature and load on the viscoelastic mechanical properties of asphalt. In this section, based on the reference temperature of 4 °C, the displacement factor values calculated in Table 5 are sequentially shifted to the left of the remaining complex modulus change curve. The principle of the displacement factor is shown in Figure 4.

**Figure 5:**
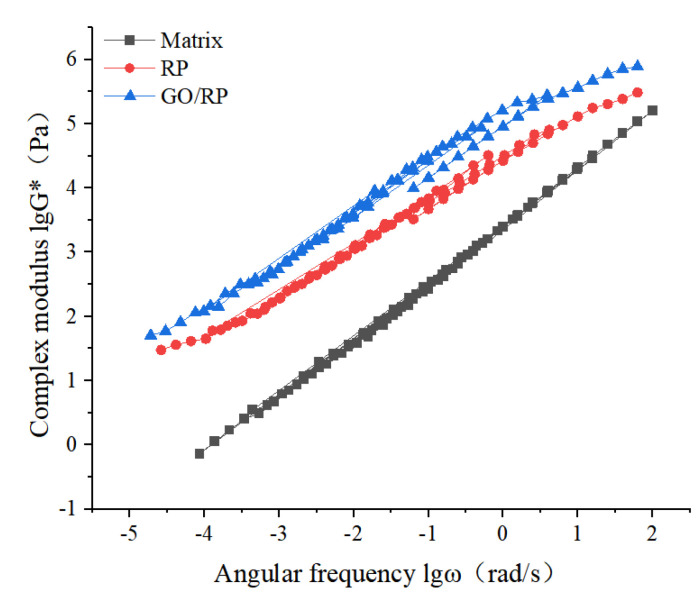
The main curves of complex modulus of three kinds of asphalt at 40 °C.

It can be seen from Figure 5 that under the conditions of high temperature and low frequency, the matrix asphalt has a small complex modulus, indicating that its high-temperature deformation resistance is weak. The complex modulus of modified asphalt is higher than that of base asphalt under the same conditions, indicating that the high-temperature rheological properties of modified asphalt are superior. For modified asphalt, the complex modulus of GO/RP composite-modified asphalt is higher than that of single RP-modified asphalt, indicating that the addition of GO can improve the resistance of asphalt to shear deformation, and thus enhance the ability of RP-modified asphalt to resist plastic deformation. Under the condition of low temperature and high frequency, the G * values of the three kinds of asphalt increase with the increase of angular frequency, and show a trend of gradually approaching. The quantitative relationship of the complex modulus of the three kinds of asphalt is GO/RP composite modified asphalt > RP modified asphalt > matrix asphalt, and the G* value of GO/RP composite modified asphalt has been in the highest position, indicating that under the condition of low temperature and high frequency, the addition of GO still significantly improves the high temperature performance of RP modified asphalt. For the whole, the composite modified asphalt has better deformation resistance at high temperature. As a whole, the composite-modified asphalt has better deformation resistance at high temperature. Here, in order to observe the frequency scanning results of three kinds of original asphalt more intuitively, the difference in the results has been obtained by corresponding means. In addition, this study adopts strain control method, so the change of stress is not considered.

### 4.2. Test of Multiple Stress Creep and Recovery

**Figure 6:**
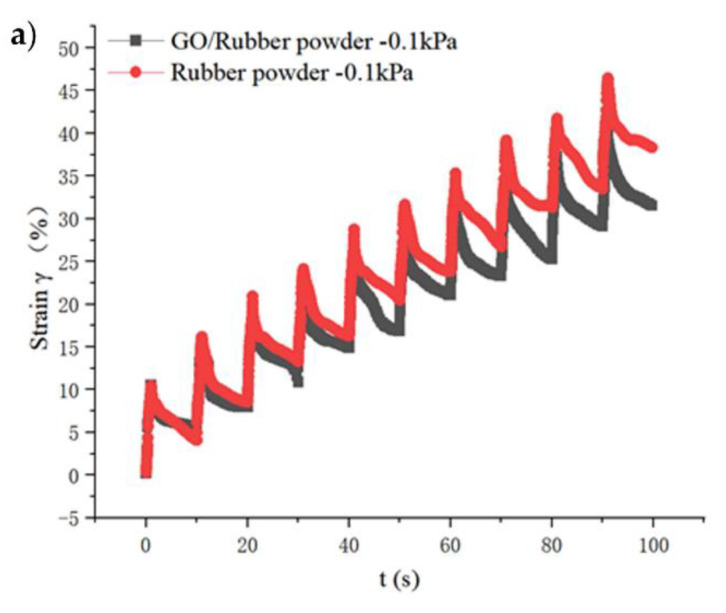

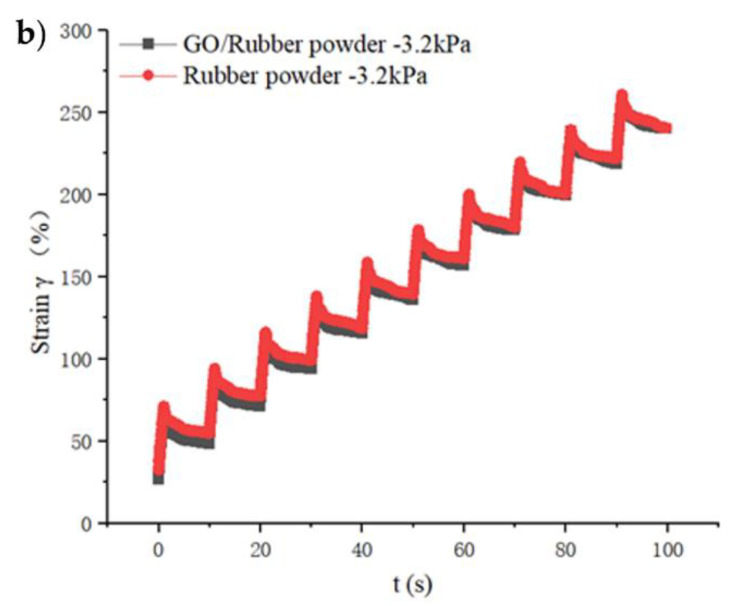
Multi-stress creep recovery test results: (**a**) results of multi-stress creep recovery test of modified asphalt (0.1 kPa stress level); (**b**) results of multi-stress creep recovery test of modified asphalt (3.2 kPa stress level).

In this section, the size of the MSCR sample is 25 mm in diameter ×2 mm in thickness, and the test temperature is 64 °C. The results are shown in Figure 6.

When the test temperature was 64 °C and the shear stress was 0.1 kPa and 3.2 kPa, the stress–strain of the base asphalt was almost rectangular, and the strain recovery rate R value was almost 0, indicating that the base asphalt had no recovery ability after loading. The unrecoverable strain accumulated after 10 test cycles is much larger than that of modified asphalt. It can be seen from Figure 6a,b that at the same temperature, the cumulative strain of GO/RP-modified asphalt at two stress levels is the smallest, indicating that the addition of GO strengthens the elastic component of the modified asphalt, thus improving its anti-plastic deformation ability at high temperature. However, when the stress increases to 3.2 kPa, the improvement effect of GO on RP-modified asphalt decreases accordingly.

**Figure 7:**
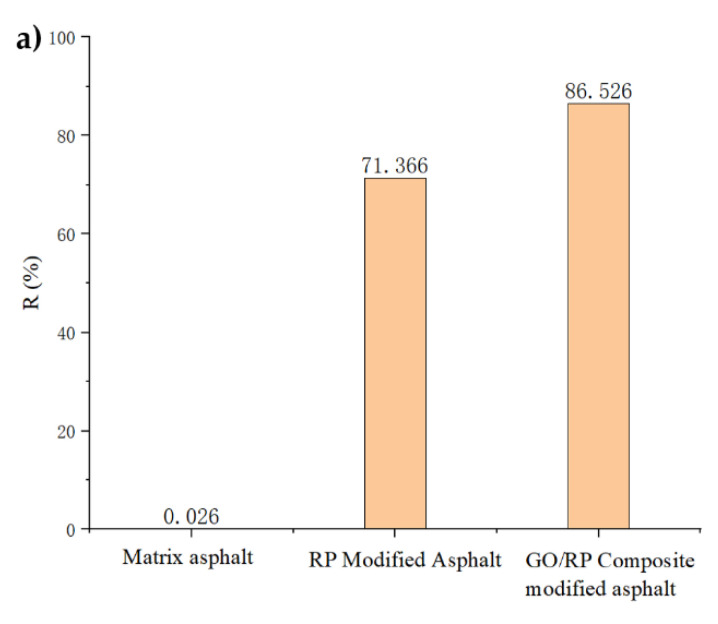

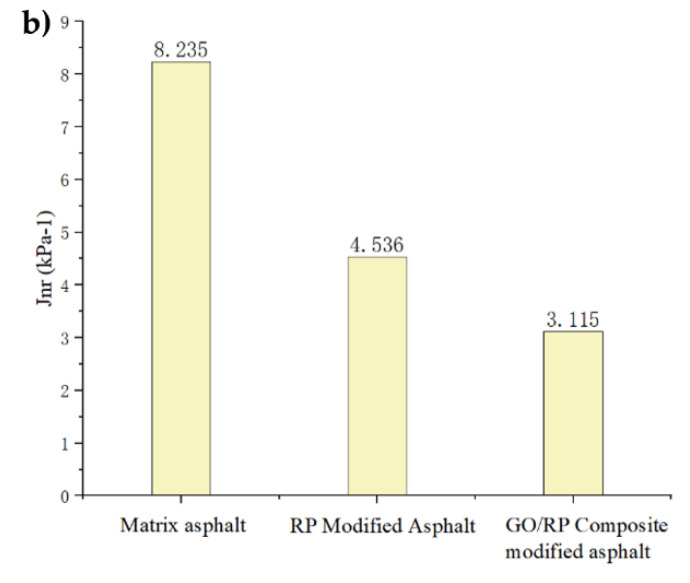
Asphalt recovery ability test results: (**a**) creep recovery rate r of different asphalt at 0.1 kPa; (**b**) unrecoverable creep compliance J_nr_ of 0.1 kPa different asphalt.

In order to show the deformation recovery ability of the three asphalts more intuitively, the R value and Jnr value of the three asphalts at the stress level of 0.1 kpa were calculated, respectively, as shown in Figure 7.

As can be seen from Figure 7, for the average strain recovery rate (R), the R value of base asphalt is very small at the stress level of 0.1 kPa, indicating that the elastic component in the base asphalt is very small and thus cannot produce recovery effect. The relationship between the R values of the three asphalts is matrix asphalt < RP-modified asphalt < GO/RP composite-modified asphalt. The addition of GO increases the R value of RP-modified asphalt by 21.2%. By observing the non-recoverable creep compliance Jnr value, it is found that the minimum value of GO/RP composite-modified asphalt is 3.115, which is 31.3% lower than that of RP-modified asphalt. Both indicate that the GO/RP composite-modified asphalt has better creep recovery ability. The analysis is that GO is a thin layer structure of carbon atoms with strong stiffness, which can improve the resistance of asphalt to deformation at high temperature, thereby enhancing the rutting resistance of its mixture. Therefore, the GO/RP composite-modified asphalt can perform well in high-temperature heavy-duty traffic applications. However, with the increase in stress, the improvement effect of GO on the recovery ability of RP-modified asphalt decreases.

### 4.3. Low-Temperature Bending Rheological Test Analysis

#### 4.3.1. Creep Stiffness S Value Analysis

**Figure 8:**
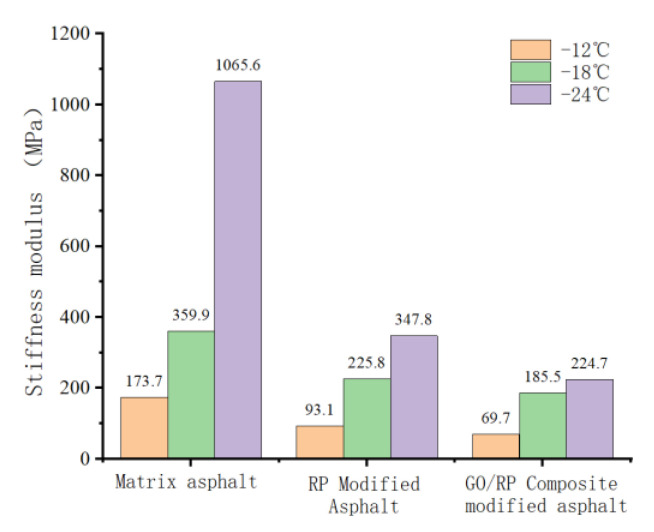
Stiffness modulus of asphalt at different temperatures.

The creep stiffness S value of the low-temperature bending creep test is automatically collected and recorded by the sensor. The creep stiffness S represents the brittleness of the asphalt. The larger the S value, the greater the low-temperature brittleness of the asphalt, and the easier it is to break. The result is shown in Figure 8.

It can be seen from Figure 8 that the stiffness modulus S value of the same asphalt increases with the decrease in temperature, among which the matrix asphalt has the largest increase, indicating that the matrix asphalt has obvious brittleness and poor deformation resistance at low temperature. At the same temperature for different asphalts, the S value of GO/RP composite-modified asphalt is the smallest, especially at −24 °C, it still meets the requirement of being less than 300 MPa, which is 78.9% lower than that of base asphalt, which is 78.9% lower than that of RP. The modified asphalt decreased by 35.4%, indicating that GO and RP as composite modifiers had better low-temperature improvement effect on matrix asphalt.

#### 4.3.2. Analysis of Creep Rate m

**Figure 9:**
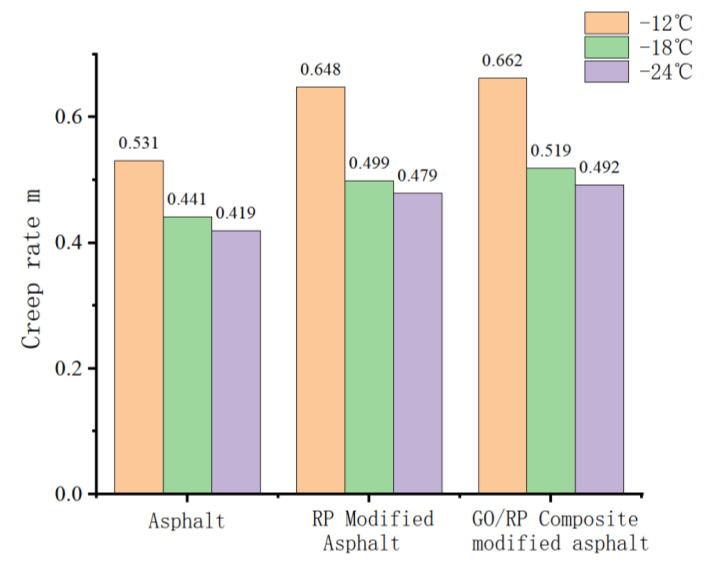
Creep rate of asphalt at different temperatures.

Creep rate m refers to the speed of creep stiffness change of asphalt trabecular specimen, which represents the ability of internal stress dispersion of asphalt. The larger the m value, the stronger the stress dispersion ability of asphalt material, the smaller the internal stress, and the better the low-temperature performance. The result is shown in Figure 9.

As can be seen from Figure 9, the m value of the same asphalt is positively correlated with temperature. The lower the temperature is, the smaller the M value is, indicating that the strength modulus of asphalt increases slowly, the stress inside it increases, and the specimen is easier to break. Under the same temperature of different asphalt, the m value of GO/RP composite-modified asphalt is the maximum, which is greater than the standard value 0.3, which again verifies that GO/RP composite-modified asphalt has good low-temperature performance.

### 4.4. Analysis of Composite-Modified Asphalt Morphology Characterization

**Figure 10:**
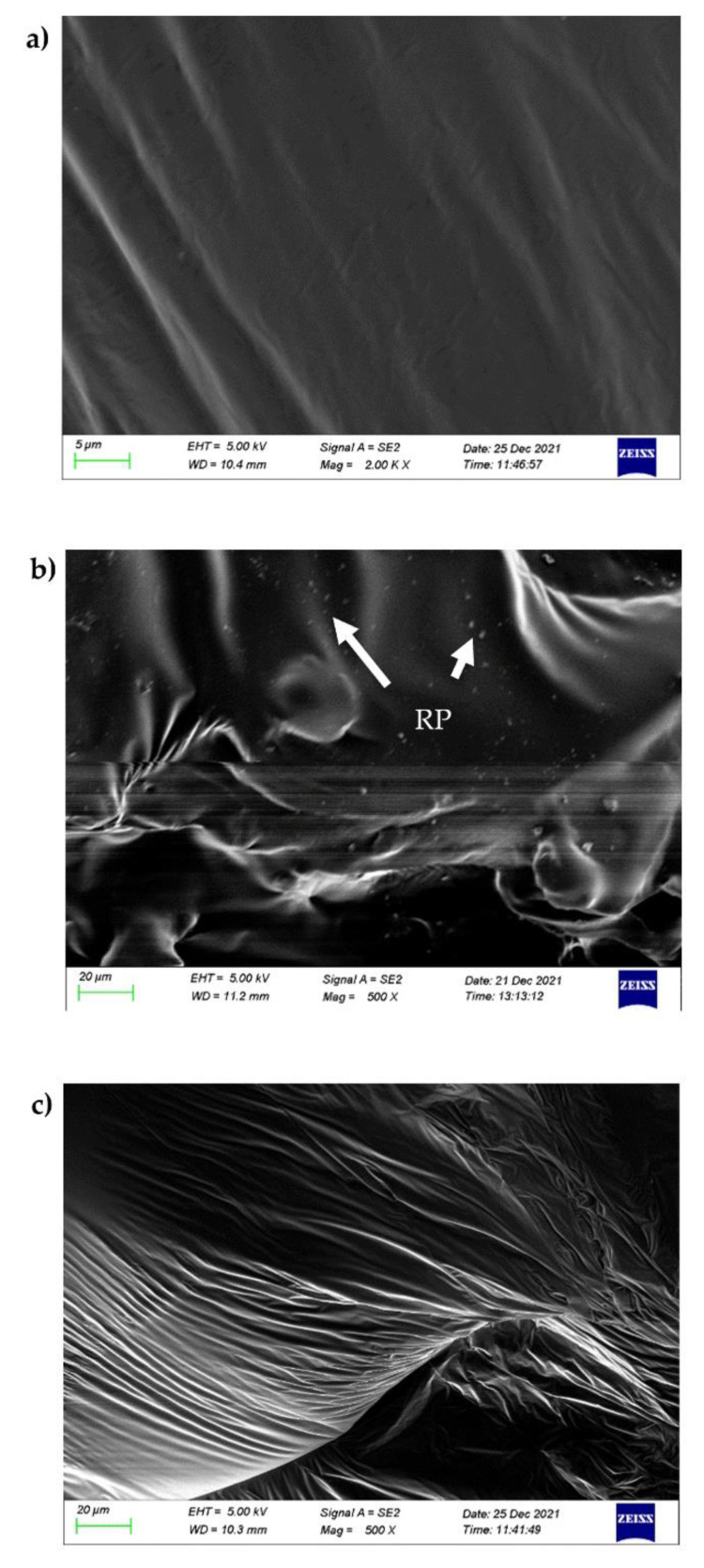
Micrograph of composite modified asphalt: (**a**). base asphalt scanning electron microscope; (**b**). SEM of modified asphalt by rubber powder; (**c**). SEM of GO/rubber powder composite-modified asphalt.

It can be seen from Figure 10a that the surface of the matrix asphalt is very smooth, with no obvious protrusions and impurities, which is a uniformly distributed asphalt phase. It can be seen from Figure 10b that the addition of RP can directly affect the microstructure of the matrix asphalt. Compared with the matrix asphalt, some of the RP-modified asphalt has obvious protrusions. The scanning electron microscope of the RP-modified asphalt shows a dense spotted phenomenon, indicating that RP cannot be well integrated with the matrix asphalt, and there is a segregation problem. Under vacuum conditions on the surface of the sample coated with a layer of conductive heavy metal particles immediately after the high-magnification observation, may be due to the conductive heavy metal particles sprayed unevenly or irradiated under the electron beam for too long. The acceleration voltage is adjusted to 5.00 kV according to the selected multiple, as shown in the figure. It can be seen from Figure 10c that the GO/RP composite-modified asphalt mainly presents an irregular wrinkle phase, and there are no raised spots. The surface of the composite-modified asphalt is smoother and the phase structure is relatively stable, which can withstand a certain amount of time. The interference of the unfavorable external environment has good stability, indicating that the composite-modified asphalt added with GO improves the segregation of RP particles in the matrix asphalt.

### 4.5. Test Analysis of Modifified Asphalt Infrared Spectrum

**Figure 11:**
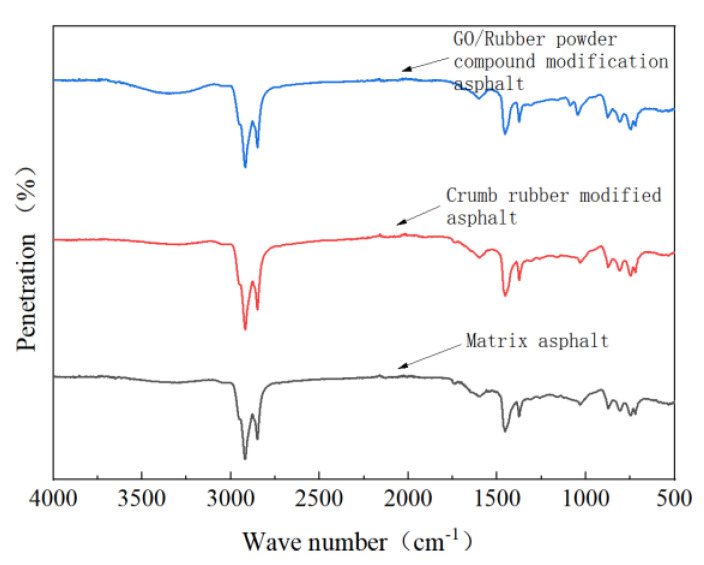
Infrared spectra of three kinds of modified asphalt.

It can be seen from Figure 11 that the infrared spectra of the three pitches have obvious characteristic peaks between the wave numbers of 2800 and 3000 cm^−1^. Taking base asphalt as an example, there are two high characteristic peaks at 2919.57 cm^−1^ and 2855.10 cm^−1^, which are generated by the stretching vibration of antisymmetric and symmetric methylene CH_2_ alkanes, respectively. It can be concluded that there are saturated hydrocarbons in asphalt. In the vicinity of 1599.63 cm^−1^, the absorption peak band with low transmittance appeared, which was generated by the vibration of C=C aromatic ring. Due to the weak vibration intensity of carbon–carbon double bond, it was in the form of a weak absorption band. The absorption peaks at 1455.68 cm^−1^ and 1375.48 cm^−1^ are similar to the two absorption peaks between 2800–3000 cm^−1^. The analysis is caused by the bending vibration of antisymmetric and symmetric methyl CH_3_ groups. The absorbance of the antisymmetric group is higher than that of the symmetric group in matrix asphalt. The weak absorption peaks at 1030.23 cm^−1^, 805.13 cm^−1^ and 722.58 cm^−1^ indicate the stretching vibration of sulfox R_1_-SO-R_2_, out-of-plane oscillation vibration of CH_2_ olefins and in-plane oscillation vibration of long-chain alkanes CH_2_ group, respectively. From the above analysis, it can be seen that the material composition of the base asphalt is relatively complex, and it contains aromatic hydrocarbon compounds after the reaction of aliphatic hydrocarbons and benzene ring substituents. It can also be seen from Figure 11 that the overall trends of the absorbance curves of the base asphalt and the RP-modified asphalt are roughly the same, indicating that RP does not have a chemical reaction in the process of modifying the base asphalt. The GO/RP composite-modified asphalt has a broadened absorption peak band in the range of 3100–3700 cm^−1^, which is due to the weak oxidation reaction between the CH functional groups in the base asphalt and GO during the preparation. In general, GO and RP mainly exist in a physically compatible form in the matrix asphalt.

## 5. Conclusions

(1) The DSR temperature scanning test results show that the addition of GO improves the physical compatibility between RP and matrix asphalt, increases the complex modulus of RP-modified asphalt, and reduces the phase angle. The rutting factor–temperature curve shows that the rutting factor of GO/RP composite-modified asphalt is the highest. By fitting the rutting factor–temperature curve, it is found that the slope |A| relationship is matrix asphalt > RP-modified asphalt > GO/RP composite-modified asphalt, indicating that GO/RP composite-modified asphalt has lower temperature sensitivity and stronger plastic deformation resistance.
(2) Through the frequency scanning test results, the complex modulus–angle frequency curve is drawn. When the temperature is between 40 °C and 88 °C, the G* values of the three asphalts are negatively correlated with the temperature. Using the time–temperature equivalence principle to construct the main curve, it can be found that the G* of the three asphalts increases with the increase in the angular frequency, and the G* relationship of the complex modulus is GO/RP composite-modified asphalt > RP-modified asphalt > matrix asphalt, indicating that the high-temperature rheological properties of GO/RP composite-modified asphalt are superior and have better deformation resistance at high temperature.
(3) According to the MSCR test data, under the condition of 64 °C, the cumulative strain of the GO/RP composite-modified asphalt at the two stress levels is the smallest. Calculating its R and Jnr at the 0.1 kPa stress level found that the addition of GO increased the R value of RP-modified asphalt by 21.2%, while the Jnr decreased by 31.3%. Both indicate that the GO/RP composite-modified asphalt has better creep recovery ability.
(4) BBR test shows that the S value of GO/RP composite-modified asphalt is the minimum at the same temperature of different asphalt, and the base asphalt decreases by 78.9% at −24 °C, and 35.4% compared with RP-modified asphalt. Moreover, the M value of GO/RP composite-modified asphalt is the maximum at the same temperature, indicating that the internal stress of asphalt is the minimum. Burgers model analysis shows that at −12 °C, GO/RP composite-modified asphalt decreases by 17.5% compared with base asphalt, and 6% compared with RP-modified asphalt, indicating that GO and RP as composite modifiers have a better low-temperature improvement effect on base asphalt.
(5) From the microscopic morphology of the two modified asphalts, it can be seen that the RP-modified asphalt with GO becomes smooth and compliant, which improves the segregation state of RP in the matrix asphalt and increases the storage stability of the rubber powder-modified asphalt. It can be seen from the infrared spectrum that the absorbance curve of GO/RP composite-modified asphalt is roughly the same as that of matrix asphalt and RP-modified asphalt. The GO/RP composite-modified asphalt has a broadened absorption peak band in the range of 3100–3700 cm^−1^, which is due to the weak oxidation reaction between the CH functional group in the matrix asphalt and GO during preparation. In general, GO and RP mainly exist in the form of physical compatibility in base asphalt. This is of great significance to the performance and mechanism of GO/RP composite-modified asphalt, and lays a theoretical foundation for future wide application.

## 6. Innovation

Compared with other materials, GO can be stably dispersed in asphalt because of its rich oxygen-containing functional groups on the surface, which is not easy to agglomerate and affect the performance of asphalt. RP-modified asphalt has better high-temperature rheological properties. After the addition of RP to matrix asphalt, the toughness of the material is improved. The mixture of the two can also complement each other and better improve the stability of the material. At present, GO and RP are rarely used as composite modifier to modify matrix asphalt at home and abroad, which is an innovation in material selection.

## Figures and Tables

**Table 1:** Basic performance index of 70# asphalt.

Test Items		Unit	Test Results	Technical Requirements	Test Method
Penetration (25 °C, 100 g 5 s)		0.1 mm	61.5	60–80	T0640
Ductility (5 cm/min, 10 °C)		cm	28.3	≤15	T0605
Ductility (5 cm/min, 15 °C)		cm	156.8	≤100	T0650
Softening point (ring and ball method)		°C	46.5	≤46	T0606
Flash point		°C	290	≤260	T0611
Density (25)		g/cm^3^	1.136	Measured value	T0603
Solubility (trichloroethylene)		%	99.86	≤99.5	T0607
After RTFOT	Quality change	%	−0.5	≥±0.8	T0610
	Penetration ratio	%	73.5	≤61	T0604
	Ductility (5 cm/min, 10 °C)	cm	8.6	≤6	T0605
	Ductility (5 cm/min, 15 °C)	cm	118.8	≤15	T0605

**Table 2:** Basic performance indexes of graphene oxide.

Purity (%)	GO Thickness (nm)	Layer Diameter (µm)	Number of Layers	Water Content (%)	Bulk Density (g/cm^3^)
98.0	1.0	12.6	1–2	1.37	0.10

**Table 3:** Fitting table of rutting factor temperature curve of three kinds of asphalt.

Asphalt Type	Fitting Formula	Correlation Coefficient R^2^
Base asphalt	lg(G*/sinδ) = −0.0526T + 3.553	0.9866
RP-modified Asphalt	lg(G*/sinδ) = −0.0482T + 3.699	0.9980
GO/RP Composite-modified Asphalt	lg(G*/sinδ) = −0.0435T + 3.538	0.9974

**Table 4:** Summary of various asphalt double logarithmic fitting curves.

Type of Asphalt	Test Temperature/°C	Fitting Curve Equation	R^2^
Matrix asphalt	40	lgG* = 0.9270 lgω + 3.3825	0.99960
	52	lgG* = 0.9266 lgω + 2.6340	0.99955
	64	lgG* = 0.8746 lgω + 1.8999	0.99691
	76	lgG* = 0.8743 lgω + 1.2975	0.99693
	88	lgG* = 0.8638 lgω + 0.7080	0.99881
RP Modified asphalt	40	lgG* = 0.6706 lgω + 4.3928	0.9932
	52	lgG* = 0.7249 lgω + 3.6495	0.9984
	64	lgG* = 0.7897 lgω + 3.0626	0.9995
	76	lgG* = 0.7049 lgω + 2.5440	0.9946
	88	lgG* = 0.6744 lgω + 2.1206	0.9834
GO/RP Composite-modified asphalt	40	lgG* = 0.6500 lgω + 4.3925	0.9824
	52	lgG* = 0.7862 lgω + 3.7325	0.9852
	64	lgG* = 0.8315 lgω + 3.0414	0.9961
	76	lgG* = 0.8037 lgω + 2.4647	0.9977
	88	lgG* = 0.7349 lgω + 1.9870	0.9916

**Table 5:** Summary of various asphalt displacement factors.

Type of Asphalt	Test Temperature/°C	lgω(G* = 1 kP, rad/s)	Displacement Factor
Matrix asphalt	40	−0.4126	0
	52	0.3950	−0.8076
	64	1.2578	−1.6704
	76	1.9473	−2.3599
	88	2.6534	−3.0660
RP Modified asphalt	40	−2.0769	0
	52	−0.8960	−1.1809
	64	−0.0793	−1.9976
	76	0.6155	−2.6924
	88	1.3040	−3.3809
GO/RP Composite-modified asphalt	40	−2.1423	0
	52	−0.9317	−1.2106
	64	−0.0498	−2.0925
	76	−0.6660	−2.8083
	88	1.3784	−3.5207

## Data Availability

All data that support the findings of this study are included within the article.
